# Notes on Liquid Articles of Dietary

**Published:** 1906-09-22

**Authors:** 


### PROGRESS IN MEDICINE AND SURGERY.

### NOTES ON LIQUID ARTICLES OF DIETARY.

**The Action of Alcohol as Medicine.**—In discussing the value of alcohol as a drug, it may be noted that as in politics so in medicine the phenomenon known as the "swing of the pendulum" is often very apparent. At one time heroic doses of alcohol are prescribed in almost every condition, at another it is termed a protoplasmic poison, and on no account to be administered to any person fighting against the processes of disease. Before considering the use of alcohol as a drug, we may shortly consider its physiological action on the healthy organism.[4] When taken by the mouth it acts as many volatile oils or pungent substances do and reflexly stimulates the heart. This action is immediate, rapid, and occurs before any absorption has taken place beyond that necessary to affect the subjacent nerve endings of the buccal mucous membrane. Secondly it acts as a vaso-dilator. The skin becomes flushed and a sensation of warmth is experienced. Thirdly, it acts as a cerebral sedative. Fourthly, owing to its fairly complete oxidation, it is, in a certain sense, a food; and lastly it stimulates gastric secretion. These physiological points will serve to place the use of alcohol as a drug on a sound basis. It may then, with advantage, be given in syncope or sudden cardiac failure as a reflex stimulant. In cases of chronic heart-disease its only value will be as a vaso-dilator, to lessen the work of the cardiac muscle when the peripheral resistance is high. With a full pulse and low tension in the arteries, alcohol can have no effect on the circulation except the transitory reflex one above described, and as a protoplasmic poison its action, at any rate in large doses, must be distinctly deleterious to the cardiac muscle. As a stomachic in cases where the gastric secretion is diminished it will also be of use; here malt liquors may be advantageous, but obviously in cases of hypersecretion its use will be contra-indicated. There is another little point to be remembered in prescribing alcohol for dyspeptics, and that is that by increasing the outflow of fluid from the blood-vessels during the process of its absorption, it will increase the amount of fluid in the stomach, and hence should not be used if that organ is dilated. In fevers alcohol has been largely used, and also largely abused. In septic cases clinical evidence is certainly in its favour, but in ordinary febrile diseases its usefulness is certainly restricted. The best guide to its administration is the pulse. As long as this is improved by the drug, so long is its use to be continued; but when the rapidity of the pulse is maintained with a falling temperature, it should immediately be discontinued. It is probable that its beneficial action is mainly due to its powers as a nervous sedative, and may be compared to the use of opium or morphia in certain cardiac conditions. It is also, of course, by reason of its vaso-dilator action, a slight antipyretic. Antipyretics are, however, by no means the "fashion" at the present time. As it is a food, albeit to a comparatively small extent, alcohol may be given in such disorders as diabetes, where there is necessarily much restriction in a particular class of foodstuffs. In gout, on the other hand, though there is no complete accord of opinion, the most generally accepted view seems to be that alcoholic liquors are mischievous. The form in which alcohol is taken is not altogether unimportant, but of still greater consequence is it to see that only sound wine and pure spirit is used for therapeutic purposes. Brandy, by reason of its high percentage of volatile ethers, has been lauded as superior to whisky, but it is assuredly a mistake to prescribe an inferior brandy, merely because it is brandy, when a sound whisky might be obtained at about the same cost.

[4] Clin. Jour., Nov. 22, 1905, p. 86.

